# Effects of ultrasonic-assisted nickel pretreatment method on electroless copper plating over graphene

**DOI:** 10.1038/s41598-022-25457-y

**Published:** 2022-12-07

**Authors:** Qingyan Peng, Xiaodong Tan, Mohanapriya Venkataraman, Jiri Militky, Wei Xiong, Arunjunai Raj Mahendran, Herfried Lammer, Pavel Kejzlar

**Affiliations:** 1 Department of Material Engineering, Faculty of Textile Engineering, Technical University of Liberec, 461 17 Liberec, Czech Republic; 2 Hubei Key Laboratory of Biomass Fiber and Eco-Dyeing and Finishing, College of Chemistry and Chemical Engineering, Wuhan Textile University, Wuhan, 430073 China; 3 Wood K plus-Competence Center for Wood Composites and Wood Chemistry, Altenberger Straße 69, 4040 Linz, Austria; 4 Institute for Nanomaterials, Advanced Technologies and Innovation, Technical University of Liberec, 461 17 Liberec, Czech Republic

**Keywords:** Materials for devices, Nanoscale materials, Soft materials

## Abstract

In this paper, copper deposited graphene was fabricated through electroless plating. A novel and facile pretreatment method is introduced based on ultrasonic treatment with nickel nano-particles as the catalytic core. This method abandons the sensitization and activation process in the traditional pretreatment that reduces the time and economic cost dramatically. The static contact angle was determined by an Olympus BX51M optical microscope. The surface morphology and plating composition were characterized via scanning electron microscope (SEM) and energy dispersive spectroscopy (EDS), the infrared radiation (IR) transmittance spectra of the copper plated graphene were measured by Fourier transform infrared spectroscopy (FTIR), the layer structure was measured by Raman spectrum, the phase identification was identified by X-ray diffraction (XRD), the thermogravimetric analysis (TGA) (Q5000 TA instruments, USA) was carried out to detect the thermal characteristics. The electrical resistivity of copper-plated graphene was performed in an especially designed apparatus. The results show that the surface of graphene is coarsened, and the size is reduced after ultrasonic treatment, which can facilitate the nucleation and fine particle distribution of metal. The electroless plated efficiency of copper of the nickel pretreatment copper-plated graphene is 64.27 wt%, higher than that of generic copper-plated graphene at 58.62 wt%. The resistivity decreases rapidly from 1.69 × 10^–2^ Ω cm of the original Gr to 0.79 × 10^–2^ Ω cm of Cu/Ni@Gr due to the large number of fine copper particles scattered around the graphene.

## Introduction

Since the discovery of graphene in 2004, a window to new technological fields has been unbarred. Due to its perfect two-dimensional (2D) lattice structure of sp^2^-bonded carbon atoms, graphene has demonstrated dazzling properties in many areas, such as unprecedented high charge carrier mobility, mechanical robustness, thermal conductivity, and the rest^1,2^. The attractive properties of graphene have made them desirable templates for “core–shell” 2D metal/graphene nanocomposites (Au, Ag, Cu, Ni, and Pd, et al.), with graphene as the matrix and metal as the outer shell^3,4^. This metallization process belongs to a sort of surface modification of graphene, not only add the surface active sites to improve the bonding strength between graphene and other materials (e.g. metal, resin, ceramic, etc.) but can maintain the superior intrinsic properties of graphene in the composites^5^. This metallization of graphene has been demonstrated to have significant potential for the fabrication of noble powdered metal/graphene composites, thus extending the application fields of graphene in corrosive resistance coating^6^, electrical contact materials^7^, electromagnetic interference^8^, active catalyst^9^.

Metal Cu has the second lowest resistivity in the natural environment and has a large free electron density (8.46 × 10^28^/m^3^). Due to its low cost, high solder ability, electro-migration resistance, and high electrical conductivity and thermal conductivity, copper has attracted the special attention of researchers for a wide range of applications. Due to its special structural properties and hydrophobic nature, the common methods such as low-temperature powder metallurgy, and chemical vapor deposition are insufficiently strong to guarantee significant adhesion between copper and graphene. Also, the tendency of agglomeration through strong Van der Waals interaction reduces the active site of graphene and curbs the formation of a copper layer with a homogenous distribution. Electroless plating is a kind of process which is widely used in modifying the surface of various materials like non-conductors, semiconductors, and metals, during which the metal ions in the solution are reduced into metal nanoparticles under a strong and appropriate reducing agent without external electrical assistance, and deposited on the surface with catalytic active sites of the matrix for forming a compact metal plating layer^6,10^. In particular, electroless plating is of great significance for improving the conductivity and service life span of some materials with high abrasive resistance requirements, such as electric contact materials. In addition, the core–shell structure composites prepared by electroless plating have good anti-fretting corrosion and anti-wear resistance properties^11^. Due to lack of catalytic sites, inert graphene does not possess autocatalytic activity, which generally needs to be pretreated by sensitization and an activation process during electroless plating, some complex technical problems still exist in these two-step processes^12^. For instance, if the activation solution contains noble metal salts (e.g. Pd, Au, Ag, etc.), the cost of noble metal would limit the wide applications of noble metal activation^13^. Stannous chloride (SnCl_2_) is frequently used in the sensitization process, and it can be hydrolyzed to form hydrogel and adhere to the surface of material easily^14^. However, the residual tin ions on the surface have an adverse impact on the uniformity and adhesion of the copper plating, resulting in an undergrade quality of electroless plating^15^. Most studies used strong acids, strong bases, strong oxides, or high temperatures to obtain new and rough surfaces, which are not environmentally safe^16,17^.

To simplify the process of electroless copper plating and bring down the economic cost, it has become an essential issue to develop non-precious metal catalysts with simple and feasible pretreatment approaches. Nano-nickel has a low price and good catalytic ability and is expected to replace noble metal as a nucleation catalyst for electroless copper plating^18^. In previous studies, although Ni^+^ was also used in the electroless copper plating process, it was usually added to the copper plating solution as an active agent after activation and sensitization^19^. Liu et al.^20^ reported a new method of surface modification pretreatment via nickel activation and ultrasonic roughening to realize electroless copper plating on the surface of mica. Ultrasound can etch the surface of the material and make it coarse and dispersive. With the successful application of ultrasound in the chemical field and other technologies for surface treatment as a particular phase of energy input, it has attracted more and more attention from researchers worldwide^21–23^.

In this paper, a simple and economical pretreatment without two-step sensitization and activation is carried out on graphene. It utilizes a nickel sulfate hexahydrate (NiSO_4_·6H_2_O) solution and supersonic wave for the pretreatment of the surface of graphene to produce metal catalytic cores and surface defects as the active site for the direct electroless copper plating. Compared to the conventional process, this approach is simplified effectively. It does not refer to any noble or poisonous metals or an expensive instrument that will dramatically reduce the process cost and cause less environmental pollution. The surface morphologies, phase composition, and electrical properties of resulting Cu/Ni@Gr composites were characterized.

## Experimental procedure

### Materials and reagents

The average sizes of graphene used in this research are 18–25 μm, purchased from supplier Epinikon a.s. as the main ingredient in this research. SnCl_2_, palladium (II) chloride (PdCl_2_), copper (II) sulfate pentahydrate (CuSO_4_·5H_2_O), EDTA Disodium Salt 2-hydrate (EDTA 2Na), potassium Sodium Tartrate (NaKC_4_H_4_O_6_·4H_2_O), formaldehyde, Potassium hexacyanoferrate(II) trihydrate (K_4_[Fe(CN)_6_]·3H_2_O), NiSO_4_·6H_2_O, sodium borohydride (NaBH_4_) were all from ALCHIMICA s.r.o. and of analytical grade without further purification. Hydrochloric acid (37%), nitric acid (70%), sodium hydroxide, and ethanol were provided by Sigma-Aldrich.

### Pretreatment of graphene powders

**Figure 1:**
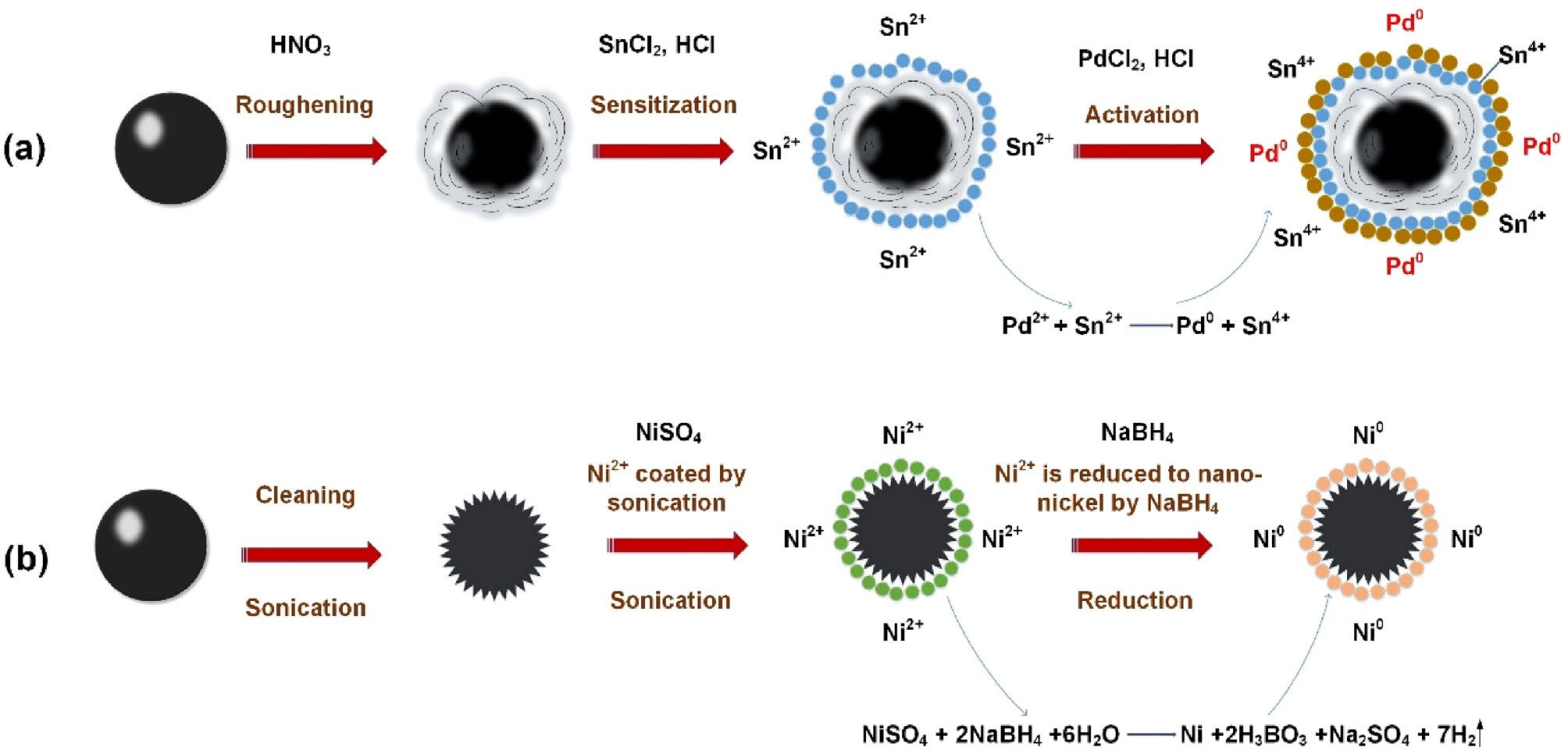
Schematic illustration of generic pretreatment for surface modification (**a**), and noble simplified palladium-free pretreatment (**b**).

The graphene powders 0.2 g were degreased in ethanol with ultrasonic cleaning for 10 min and filtered off by vacuum. The NiSO_4_·6H_2_O (20 g/L) was dissolved in deionized water with mechanical agitation. After degreasing, the graphene was added at room temperature with probe-type ultrasonic processing for 12 min and then transferred to mechanically stirring for 30 min. During this procedure, the surface coarseness of graphene and the adsorption of nickel ions can be accomplished simultaneously and efficiently. After filtration, the graphene deposited with nickel ions was added into a NaBH_4_ solution (0.5 mol/L) with mechanically stirring to reduce the Ni^2+^ to Ni^0^ (shown in Fig. 1b).

To compare, we modified the surface of graphene according to the generic method. 0.2 g graphene was mixed with HNO_3_ at room temperature for 30 min to coarse. And then transferred into an SnCl_2_ (30 g/L) and HCl (60 mL/L) mixed solution by mechanically stirring for 15 min. After that, the sensitized graphene was blended with the solution of PdCl_2_ (0.25 g/L) and HCl (10 mL/L) by agitating for 15 min to actualize activation (shown in Fig. 1a).

### Electroless copper plating

The activated graphene was taken into an electroless copper plating bath in 100 mL quantity. The plating bath containing CuSO_4_·5H_2_O, EDTA 2Na., NaKC_4_H_4_O_6_·4H_2_O, K_4_[Fe(CN)_6_]·3H_2_O and NaOH, the NaBH_4_ as reducing agent. The detailed composition of the electroless copper plating bath is listed in Table 1. The process was carried out at room temperature and pH 12.5–13 under mechanically stirring until the end of the reaction (a large amount of gas would be generated during the reaction). The copper-plated graphene was obtained after washing with deionized water several times and drying in the oven at 80 °C for 12 h. The graphene prepared with various methods were named Gr, Ni@Gr, Cu/Ni@Gr, and Cu@Gr, respectively.

**Table 1:** Chemical composition of the electroless copper plating bath.

Role in the bath	Chemical	Concentration
Main salt	CuSO_4_·5H_2_O	15 g/L
Complexing agent	EDTA 2Na	14.5 g/L
	NaKC_4_H_4_O_6_·4H_2_O	14 g/L
Buffering agent	K_4_[Fe(CN)_6_]·3H_2_O	1 g/L
Reducing agent	NaBH_4_	2 g/L
PH adjuster	NaOH	Proper amount

### Morphological observations

The synthesized graphene composites were characterized by UHR-SEM Zeiss Ultra Plus with an accelerating voltage of 2 kV equipped with an Energy Dispersive X-ray (EDX) spectrometer Oxford X-max 20. EDX analysis of the resulting composites was performed at 10 kV accelerating voltage to confirm the elemental configuration of the deposited materials on the surface of graphene, all the samples were gold-sputtered and carefully handled to avoid contaminations. Malvern zetasizer nanoparticle characterization system was used to study the particle size distribution. The static contact angle() was determined to describe the hydrophilicity improvement of graphene after ultrasonication treatment by an Olympus BX51M optical microscope fitted with an Olympus E-510 digital single-lens reflex camera. The electroless plated efficiency of copper (${m}_{Cu}$) on the graphene was measured via a chemical oxidation–reduction approach. The composites were heated up to 900 °C in an air atmosphere for 2 h, removed to a dryer for cooling for 30 min, and then weighed the mass and repeated the above process until the weight error was less than 0.001 g. After oxidation at high temperature, graphene was oxidized into gases, and the mass of residual ash can be negligible. At the same time, the copper is transferred into copper oxide and calculated according to the following equation^24,25^:

1 $${m}_{Cu}=\left(\frac{{M}_{Cu}}{{M}_{Cu}O}\right)\times \left(\frac{{m}_{CuO}}{{m}_{composite}}\right)\times 100\%,$$

where ${M}_{Cu}$ is the relative atomic mass of Cu, ${M}_{Cu}O$ is the relative molecular mass of CuO, ${m}_{CuO}$ represents the mass of copper oxide and ${m}_{Cu\&C}$ represents the mass of the composite.

### Spectroscopic analysis

The Fourier transform infrared spectroscopy (FTIR) spectra of the copper-plated graphene were measured by Nicolet iZ10 Thermo Scientific infrared spectrophotometer (KBr pellets) in the range of 4000–400 cm^-1^. The spectra were corrected by atmospheric and baseline correction in the Omnic software. A DXR™ Raman microscope (Thermo Scientific, USA) with a 532 nm laser and full range grating with 900 lines/mm was used. Samples were placed under the lens of an Olympus optical microscope at 10 × magnification. The spectra were obtained in the range of 3500–50 cm^−1^. X-ray diffraction (XRD) was carried out using X׳Pert PRO MPD diffractometer (PANalytical) in Bragg–Brentano geometry equipped with a Co X-ray tube (iron filtered CoKα radiation: λ = 0.178901 nm), all materials were prepared on zero-background Si slide and measured in 2θ range 5°–105° (resolution 0.017° 2θ).

### Thermal and ohmic heating behavior

Thermogravimetric analysis (TGA) (Q5000 TA instruments, USA) was carried out to detect the thermal characteristics of copper plated graphene in the temperature range of 25–800 °C at a heating rate of 10 °C/min. The measurements were performed under a nitrogen atmosphere at a flow rate of 25 mL/min. The ohmic heating behavior of samples was estimated using an electrical power supply (TIPA SP3010) with a clamping distance of 0.5 cm, and an infrared camera (FLIR-E6390, FLIR SWEDEN) was used to monitor the temperature change on the sample surface. Before testing, the particles were compressed into a circular sheet with a diameter of 1 cm by a mold.

### Electrical resistivity

**Figure 2:**
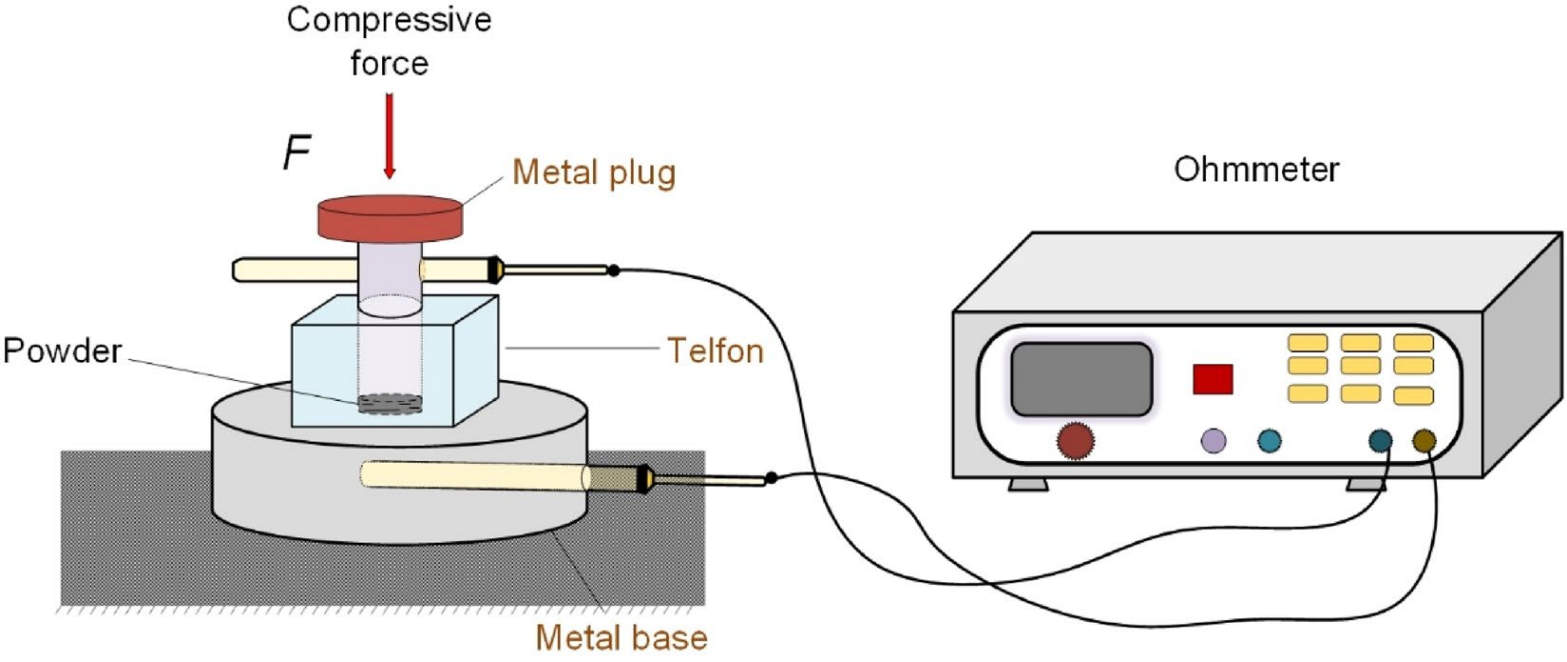
Schematic of the experimental apparatus for the determination of the electrical resistance of powders.

The electrical resistivity of copper-plated graphene was performed in an especially designed apparatus, which is schematically shown in Fig. 2. The apparatus consists of a thick isolating Telfon die, a close-fitting copper plug which is allowed to move down in the cylinder, close the compression chamber. After filling the chamber with a certain amount of graphene, the porosity of the powders is diminished by increasing the pressure through the upper plug. The effective resistivity of the graphene ($\mathrm{\rho E}$) can be calculated from the electrical resistance (${\mathrm{R}}_{measured}$), using the following equation^26^:

2 $$\mathrm{\rho E}={\mathrm{R}}_{measured}\left(\frac{{S}_{N}}{h}\right),$$

where the ${\mathrm{R}}_{measured}$ is the resistance obtained from the measurement, ${S}_{N}$ is the die orifice cross-section (0.5 cm of diameter), and $h$ represents the powder column height.

### Ethics declarations

Manuscripts reporting studies that do not involve human/animal participants, human/animal data, or human/animal tissue.

## Results and discussion

### Wettability of graphene

**Figure 3:**
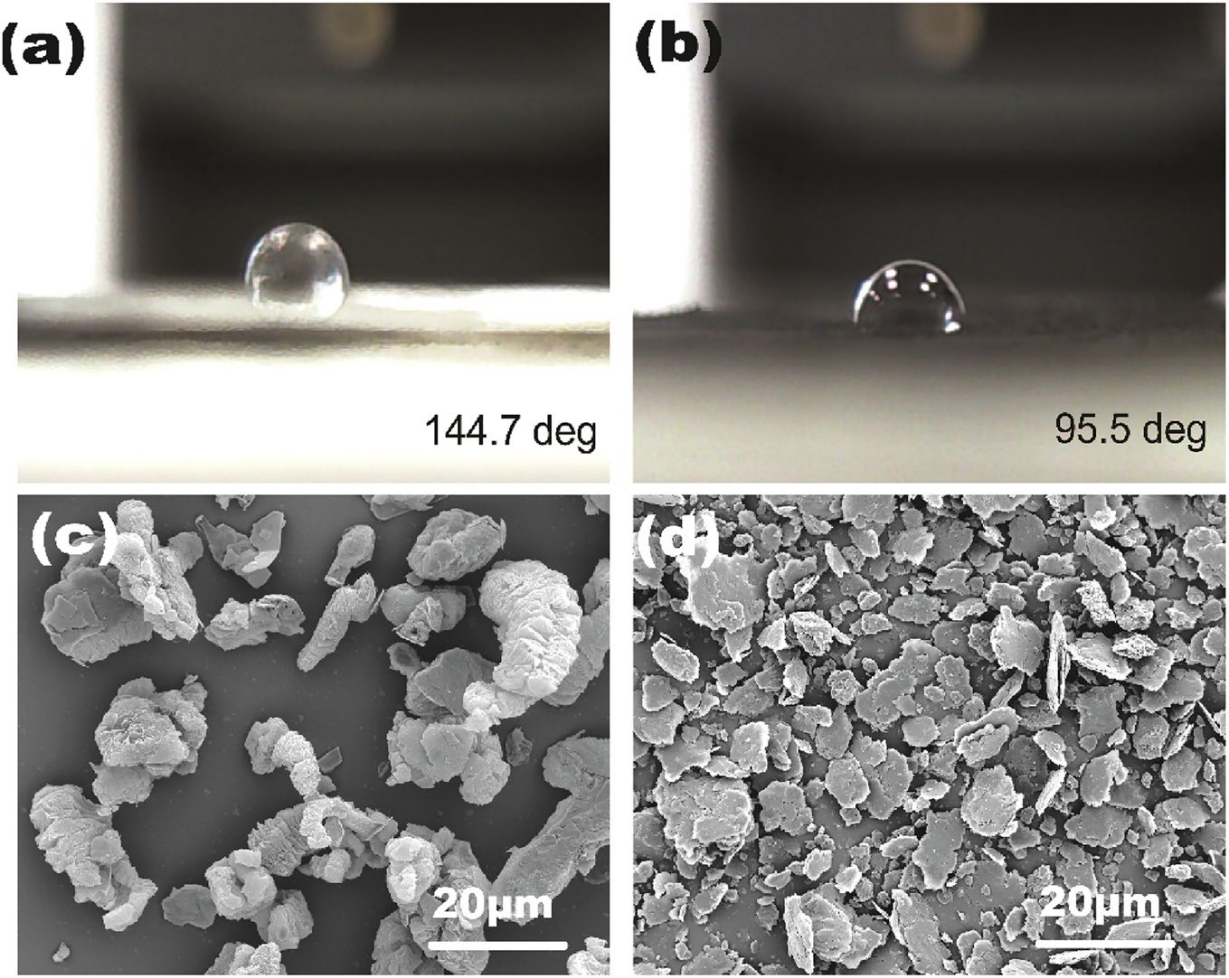
Side-view optical images of water-drop on (**a**) original graphene film and (**b**) ultrasonic treated graphene film. SEM images of the original graphene (**c**), after ultrasonic treated graphene (**d**).

The ultrasound cavitation in low frequency can etch the surface of the metal and make it rough. Figure 3a,b shows the optical images of water-drop on original graphene film and ultrasonic treated graphene film. The contact angle between pristine graphene and water is around 144.7°, while the contact angle between ultrasonic treated graphene and water is 95.5°. This proves that graphene made a transition from hydrophobic to hydrophilic property after the treatment through ultrasonication. Ideally, graphene is a monolayer of carbon atoms tightly bound in a hexagonal honeycomb lattice. However, it is difficult to exfoliate it into a monolayer structure in practical commercial production. During ultrasonic pretreatment, the bulk graphene consists of multiwall flake graphene via weak Van der Waals force, which is easily torn off from the bulk surface by ultrasonic vibration and generates a large free surface and better dispersion. As seen in Fig. 3c,d, the graphene flake obtained good exfoliation and the size was significantly reduced after ultrasonic pretreatment. The improvement of wettability would contribute to the deposition of nickel and facilitate the subsequent electroless plating on graphene.

### SEM/EDX analysis

**Figure 4:**
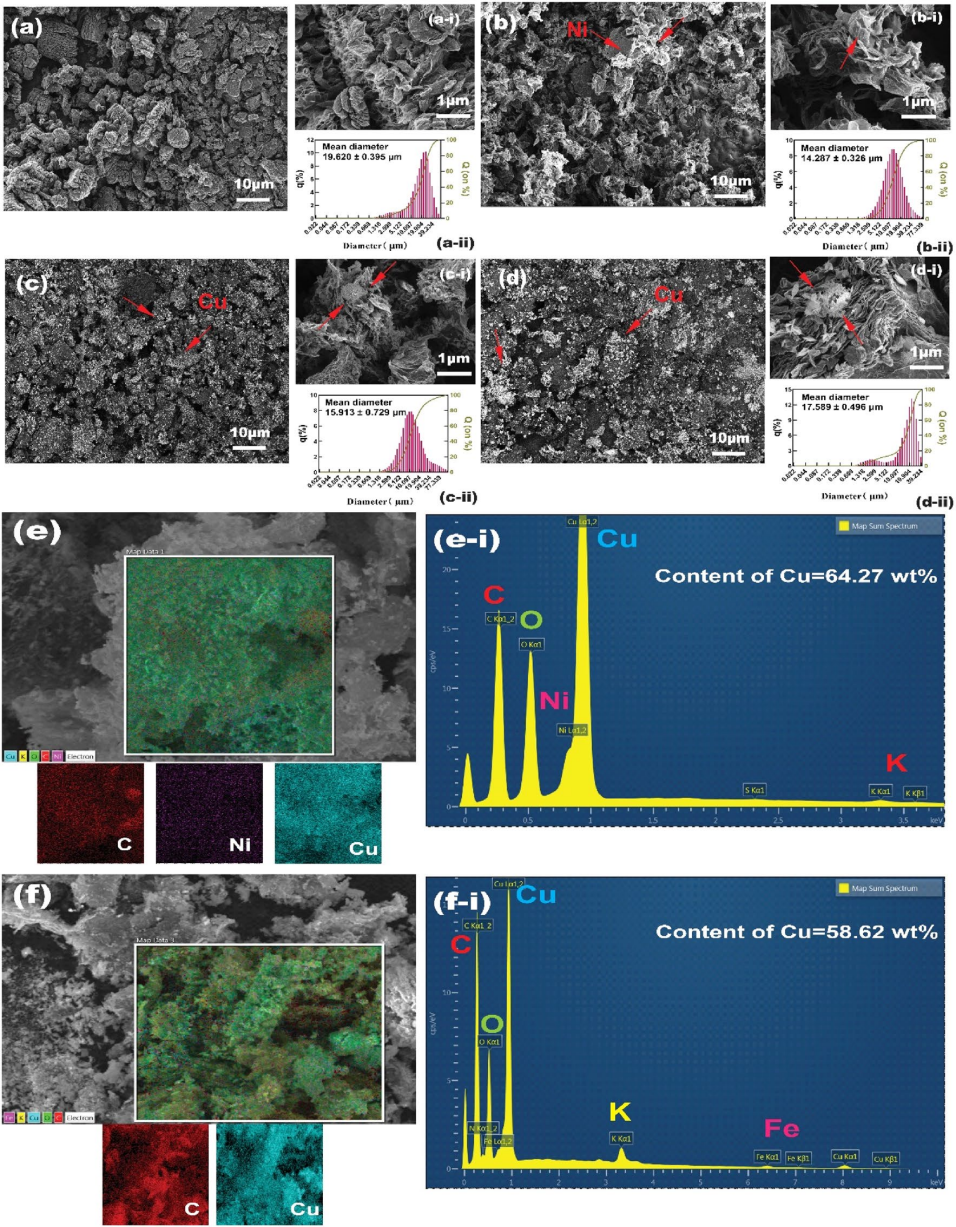
SEM images of the original graphene (**a**), nickel pretreated (**b**) and after nickel pretreatment copper-plated graphene (**c**), and generic copper-plated graphene (**d**); The high magnification images (**a-i**)–(**d-i**) and the size distribution (**a-ii**)–(**d-ii**) of them, respectively. Elemental mapping of nickel pretreatment copper-plated graphene (**e**) and generic copper-plated graphene (**f**); EDS spectra of nickel pretreatment copper-plated graphene (**e-i**) and generic copper-plated graphene (**f-i**).

Figure 4 shows SEM images of graphene particles before (**a**) and after (**b**) nickel pretreatment under ultrasonic sound. The raw graphene particles have a stacked layered structure that the surface is comparatively smooth. While the nickel pretreated graphene shows a crimping and rough surface, the particles changed into angular ones. Therefore, the specific surface of graphene increased. And the average particle size also reduced from 19.620 ± 0.395 μm of the raw graphene to 14.287 ± 0.326 μm after pretreatment, indicating that the nickel pretreatment not only gave it more metal catalytic cores but also reduced the size of graphene. As shown in Fig. 4a,d, a layer of agglomerate copper nanoparticles was deposited on the surface of graphene, which indicated the success of electroless plating. Some research on in situ chemical reduction of graphene oxide proved that metal ions tend to nucleate at the sites of functional groups^27^. But for graphene, very few functional groups take part in chemical reactions during the electroless plating process. Metal ions are inclined to aggregate and nucleate at the defects and activated sites where nucleation energy is low on the surface of graphene^28^. The typical cauliflower structure of copper deposition is observed at the edge of graphene with higher magnification (Fig. 4c-i,d-i), which confirms the tendency of metal aggregation and nucleation described above. The particle size distributions of nickel pretreatment copper-plated and generic copper-plated graphene are shown in Fig. 4c-ii,d-ii. It is considerably clear that the particle size of the latter is larger than that of the former since the mechanical agitation is not able to as effective as an ultrasonic shear force in exfoliating graphene.

Figure 4e,f show the elemental mapping images of copper and other elements present on the surface of the copper-plated graphene. Regarding the nickel pretreatment method, the distribution of elements within the white box shows that the graphene surface contains both copper and nickel, indicating that copper particles were successfully deposited and densely distributed on the graphene. The corresponding EDS spectra of nickel pretreatment copper-plated graphene are shown in Fig. 4e-i; the copper content on the graphene is about 64.27 wt% (the mass change of Cu/Ni@Gr is almost the same as that of Gr after high-temperature heating, so the mass of nickel can be ignored by chemical oxidation–reduction approach). Figure 4f-i shows the copper content of generic copper-plated graphene is around 58.62 wt%, which is a little bit lower than nickel pretreatment copper-plated graphene. Besides, the potassium and iron elements detected in the EDS spectra are mainly from the stabilizers and complexing agents of the plating solution.

### FTIR, XRD and Raman spectroscopy of copper-plated graphene

**Figure 5:**
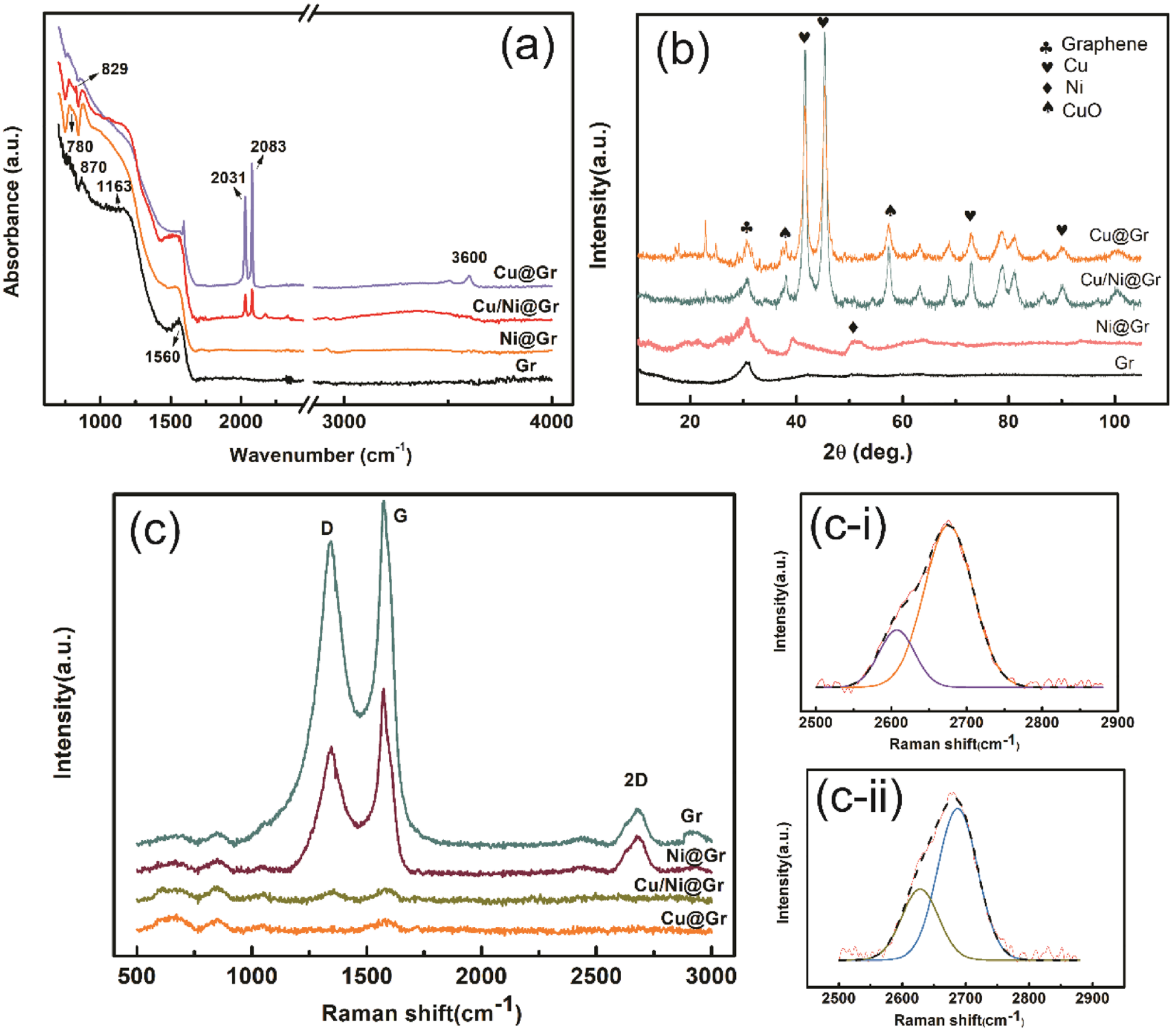
FTIR spectrum of the copper-plated graphene as a function of wave number (**a**). XRD patterns of Gr, Ni@Gr, Cu/Ni@Gr, and Cu@Gr (**b**). Raman spectra of copper-plated graphene (**c**) and the deconvoluted 2D peak of Gr (**c-i**) and Ni@Gr (**c-ii**).

FTIR spectroscopy is presented as a non-destructive analytical technique to determine the chemical bonds between atoms in materials. FTIR spectra in Fig. 5a shows the presence of the absorption peak observed at 870 cm^−1^ for original graphene, which can be attributed to the bending or deformation vibration of C–O–C bonds. The peak at 1163 cm^−1^ and 1560 cm^−1^ corresponds to the stretching vibration of C–O carbonyl groups and C=C aromatic groups, respectively. In the case of the nickel functionalized graphene, there are new signals occur in the FTIR spectra, which are observed at 780 cm^−1^ corresponding to C–H out-of-plane bending vibration, indicating that there are new chemical bonds generated between nickel nanoparticles and graphene, rather than simple physical adsorption. By comparison of the FTIR spectra of Cu/Ni@Gr and Cu@Gr, it is obvious that two new sharp peaks appear at wavelengths between 2000 and 2100 cm^−1^ for both Cu/Ni@Gr and Cu@Gr. The medium peak observed at 2031 cm^−1^ was assigned to the stretching vibration of C=C=C, and the strong peak at 2083 cm^−1^ corresponds to the asymmetric stretch of C–H. The results indicate that the copper layer has been plating onto the surface of particles successfully. It should be noted that the spectrum of Cu@G shows that O–H stretched and vibrated at 3600 cm^−1^, which probably occurred because of the hydroxide form during the sensitization and activation process. Moreover, in the case of Cu/Ni@Gr, a peak at wavenumber 829 cm^−1^ is likely to indicate the presence of Cu-OH vibration modes.

Figure 5b demonstrates XRD patterns of graphene with different electroless copper plating. As shown in Fig. 5b, one peak at 2θ = 30.9°correspond to the (002) crystal planes of the graphene is observed in all the patterns. Three peaks in the XRD pattern of Cu/Ni@Gr and Cu@Gr, located at 2θ = 42.9°, 45.8°, 73.0° and 90.1°, corresponding to the (111), (200), (220) and (311) planes of copper, indicating the successful coating of copper on the surface of the graphene. Besides, the width of the above diffraction peaks is broadened, which means the reduced copper particles are nanoscale and of low crystallinity^29^. The diffraction peaks seen at 2θ = 38.1° and 57.3° were assigned to CuO which was attributed to the following chemical side reaction during electroless copper plating. In addition, the (011) characteristic reflection peak of nickel, located at 2θ = 44.5° is observed in the Ni@Gr, whereas it disappeared in the Cu/Ni@Gr. This might be due to the copper layer deposited on the surface of Ni@Gr, where the nickel had functioned as nucleate points. And the peak observed at 2θ = 63.4°(440) could be attributed to face-centered cubic Fe_3_O_4_, which came from residual K_4_[Fe(CN)_6_]·3H_2_O during copper plating process.

Raman spectroscopy is an ideal tool to analyze the quality, thickness, and uniformity of graphene and its derivatives in detail^30^. Figure 5c shows the Raman spectra of original and electroless plated graphene particles. Three typical characteristic peaks at about 1348.50 cm^−1^, 1581.66 cm^−1^, and 2680.28 cm^−1^ are displayed in original graphene and nickel functionalized graphene, which corresponds to D, G, and 2D peaks, respectively. The G peak (1580–1590 cm^−1^) is the main characteristic peak of graphene, mainly formed by the in-plane vibration of carbon atoms, which is very sensitive to the number of graphene layers. As the number of layers increases, the G peak moves toward the direction of the lower position, indicating the weakening of bond energy between graphene layers. An empirical formula for calculating the number of graphene layers^31^:

3 $${\mathrm{W}}_{G}=1581.6+11/(1+{\mathrm{n}}^{1.6}),$$

where ${\mathrm{W}}_{G}$ is the position of the peak, and $\mathrm{n}$ is the layers of graphene. According to the formula mentioned above, it is possible to estimate that the number of layers of graphene is around 26 layers. The D peak (1270–1450 cm^−1^) is generally considered to be the disordered vibration peak of graphene, which is caused by the lattice movement away from the center of the Brillouin region and is used to characterize defects in the graphene specimen. The 2D peak (2680–2700 cm^−1^) is a biphonon resonant Raman peak, which can also be used to evaluate the number of graphene sheets. It is known that the electronic dispersion of multilayer and monolayer graphene is different, leading to the obvious difference in Raman spectra^32^. From Fig. 5c-i,c-ii, pure Gr and Ni@Gr peaks can be split into two Lorentzen peaks, indicating that the graphene used in the research is defined as multilayer graphene. And the intensity ratios I_2D_/I_G_ of Gr and Gr/Ni are about 0.24 and 0.29, respectively, compared with that of the single-layer graphene is around 2 and bi-layer graphene is around 1. However, after electroless copper plating onto the surface of the particles, the characteristic peak of grapheme disappeared, indicating that the graphene had been deposited with copper nanoparticles.

### Thermal stability analysis of copper-plated graphene

**Figure 6:**
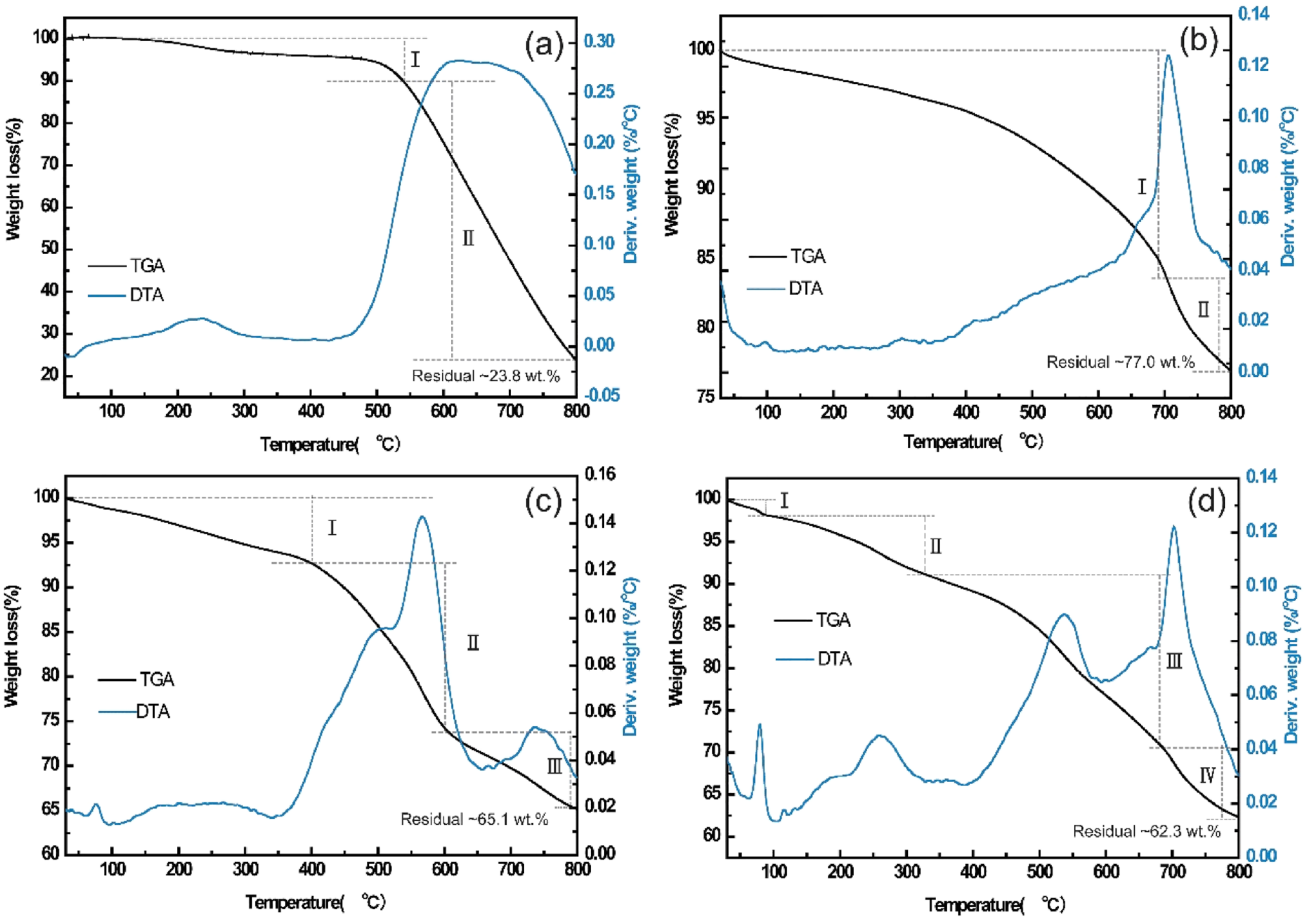
TGA and DTA curves of (**a**) Gr, (**b**) Ni@Gr, (**c**) Cu/Ni@Gr, and (**d**) Cu@Gr.

Thermal stability measurement is a versatile approach to characterize the property of the plating layer, it describes the thermal resistance of the compounds by heating them. Thermal stability can be analyzed by thermogravimetric analysis (TGA) and differential thermal analysis (DTA). Figure 6 shows the TGA and DTA curves of original graphene, nickel deposited graphene, copper deposited graphene, and nickel/copper deposited graphene at a temperature from 30 to 800 °C. The graphene exhibits two distinct weight loss regions. The first slight mass loss ended at ~ 520 °C and was mainly due to the loss of physisorbed water, water of crystallization, and some sulfur. The second region, which exhibits a significant mass loss is detected only when the graphene sample is heated up to 520 °C, which can be attributed to the decomposition of the graphene as reported in literatures^33,34^. It coincides with two wide peaks of the DTA curve in Fig. 6a at approximately 235 °C. and 608 °C, respectively. The TGA curve of nickel-deposited graphene in Fig. 6b shows a gradual weight loss occurred up to 693 °C, which can also be explained based on the decomposition of the graphene. And the slower rate of degradation compared to original graphene may be due to the protective effect of the surface nickel layer. Then, a drastic weight loss up to 800 °C and the sharp peak at 709 °C in the DTA curve reflect the decomposition of residual NiSO_4_ to NiO and SO_3_. As for Fig. 6c, The Cu/Ni@Gr shows three weight loss stages in atmospheric nitrogen. Stage I, at around ~ 400 °C, a weight loss of 8.5% can be related to burning the amorphous carbon and graphene with defects. Stage II, between 400 and 605 °C, a weight loss of about 20% can be observed, at this temperature, part of the graphene gets burned. And 7.4% weight loss occurs between 605 and 800 °C during Stage III, compared with Ni@Gr, the residual weight of Cu/Ni@Gr decreases from 77.0 to 65.1 wt%, this may be due to the catalytic nature of copper promoting graphene combustion^35^. Figure 6d demonstrates the thermal stability profile of Cu@Gr, which exhibits four weight loss stages, coincides with four exothermic peaks of the DTA curve, and the residual weight is about 62.3 wt%.

### Ohmic heating behavior of copper-plated graphene

**Figure 7:**
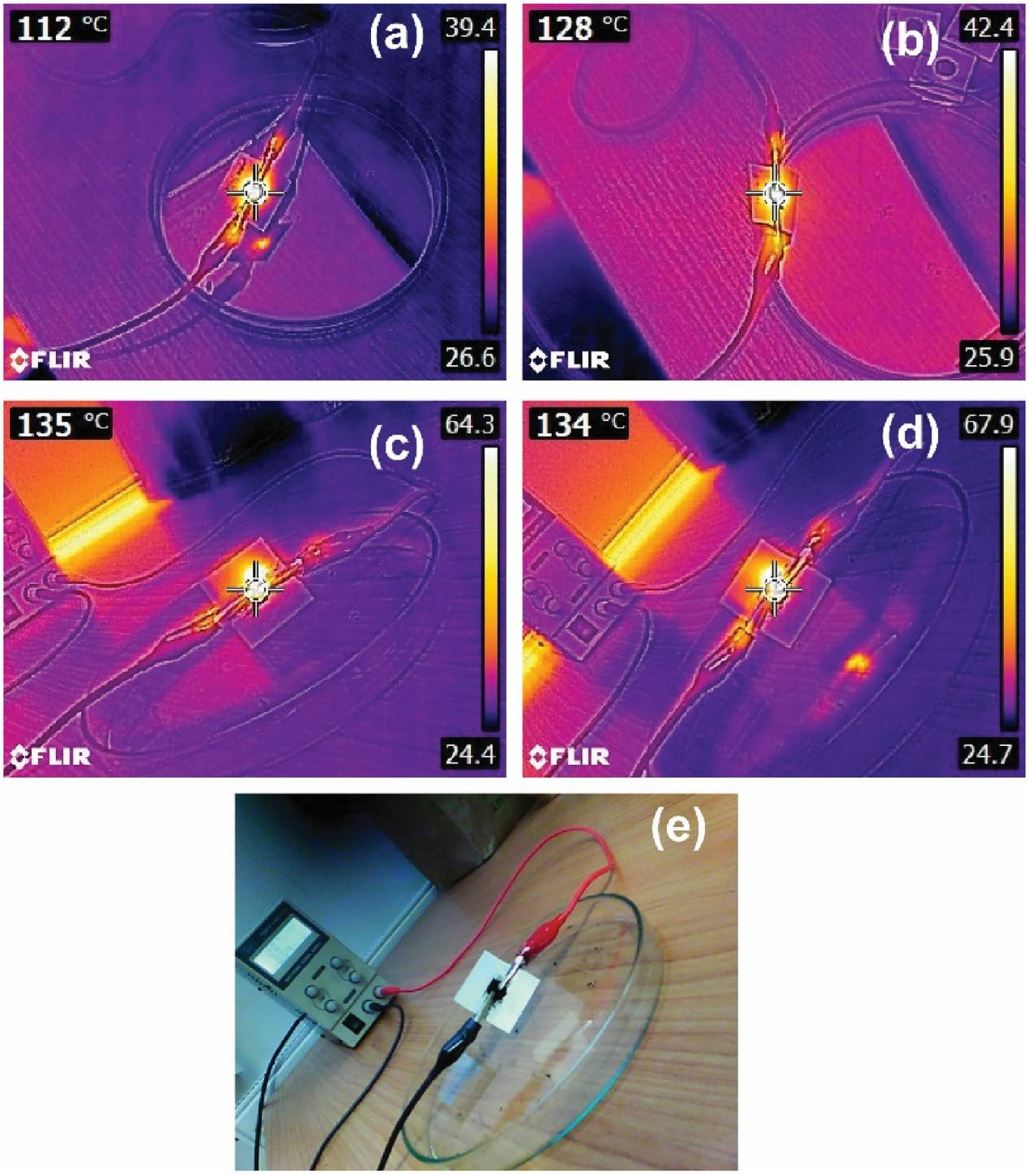
IR thermal camera images of (**a**) Gr, (**b**) Ni@Gr, (**c**) Cu/Ni@Gr, and (**d**) Cu@Gr at 10 V, 1 min, and (**e**) digital photo of the test configuration.

Figure 7 shows IR thermal images of graphene before and after being plated with metal obtained under a 10 V voltage in an ambient atmosphere. The image of Cu/Ni@G and Cu@Gr display a bright emission area with the highest temperature of ∼ 135 °C and ∼ 134 °C, whereas Gr shows the highest temperature of ∼ 112 °C only. This obvious distinction could be traced back to Joule's law which can be written as Eq. (4)^36^:

4 $$\mathrm{P}=\frac{{v}^{2}}{R}t,$$

where $\mathrm{P}$ is the amount of heat generation (in Joules), and $\mathrm{t}$ is the time duration. According to the results of electrical resistivity testing, since Cu/Ni@Gr and Cu@Gr exhibit a lower resistance, they should exhibit a higher temperature at the same applied voltage. This can be attributed to the fact that Cu has the second lowest resistivity in the world and has a large free electron density (8.46 × 10^28^/m^3^), after electroless copper plating on graphene by ultrasonic-assistant, the resistivity of the samples has been greatly improved.

### Electrical resistivity of copper-plated graphene

**Figure 8:**
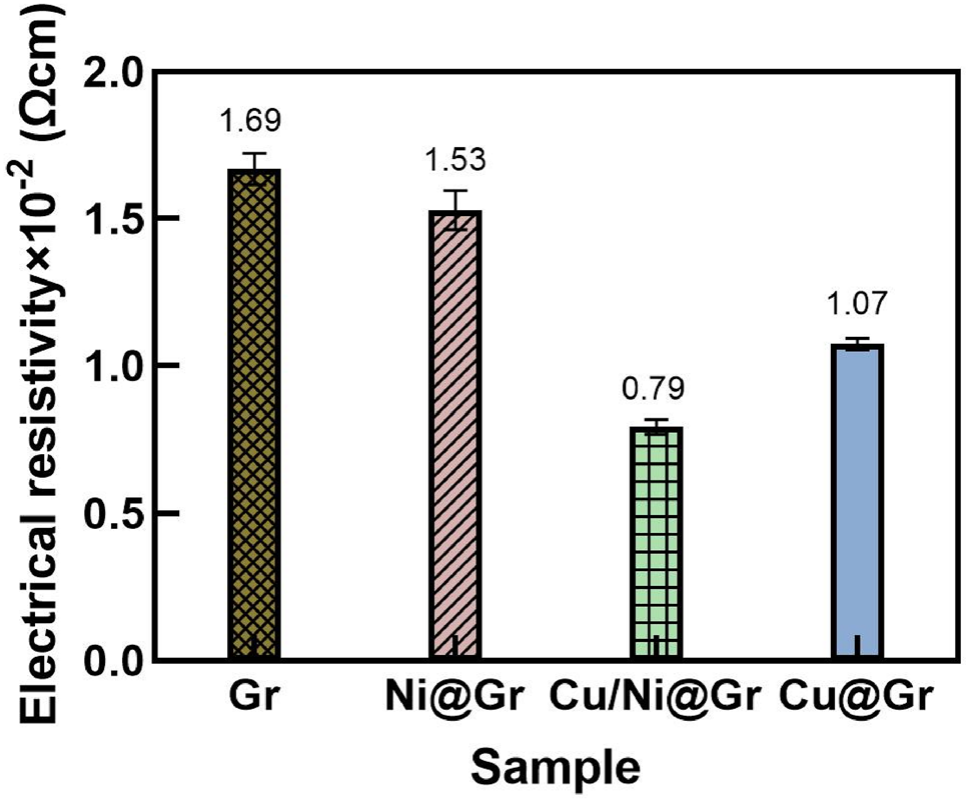
Electrical resistivity of (**a**) Gr, (**b**) Ni@Gr, (**c**) Cu/Ni@Gr, and (**d**) Cu@Gr.

Figure 8 shows the value of electrical resistivity of particles with different treatment processes during the electroless copper plating. The resistivity decreases rapidly from 1.69 × 10^–2^ Ω cm of the original Gr to 0.79 × 10^–2^ Ω cm of Cu/Ni@Gr and 1.07 × Ω cm of Cu@Gr due to the large number of fine copper particles scattered around the graphene, which can be beneficial to build a high qualified conductive path. Besides, the smaller the size of graphene, the larger the specific surface area and the better the surface adhesion of particles, which is beneficial to the formation of copper crystal nuclei and the growth of crystalline grains. Compared to Cu@Gr, Cu/Ni@Gr has a smaller particle size, leading to a better-plated efficiency of copper on its surface. The above phenomenon is in good agreement with the SEM observations. Novoselov et al.^37^ found that the electrical resistivity of monolayer graphene is approximately 1 μΩ cm, which is fairly lower than that of the above results. This could be explained as follows, the electrical resistivity of graphene increases with the increasing thickness (a similar behavior can be observed for graphite) due to the inhibited carrier mobility. This is consistent with the results obtained by SEM and Raman analysis that the graphene used in this research has multilayers and contains more defects. And contact resistance between particles must be taken into account in the case of granular materials^38^.

## Conclusion

The contact angle of ultrasonic treated graphene is 95.5°, much lower than that of pristine graphene 144.7°, demonstrating that graphene made a transition from hydrophobic to hydrophilic properties after the treatment through ultrasonication. The EDS, FTIR, Raman, and XRD spectra show that copper particles were successfully deposited and densely distributed on the surface of graphene. Compared with Cu@Gr, the copper content on the Cu/Ni@G increases from 58.62 to 64.27 wt%. Due to the deposition of the metal layer, the thermal stability of copper-plated graphene has been greatly improved. Thermal IR thermal images of Cu/Ni@G and Cu@Gr display the highest temperature of ∼ 135 °C and ∼ 134 °C, whereas Gr shows the highest temperature of ∼ 112 °C only. The resistivity decreases rapidly from 1.69 × 10^–2^ Ω cm of the Gr to 0.79 × 10^–2^ Ω cm of Cu/Ni@Gr and 1.07 × Ω cm of Cu@Gr, since the large number of fine copper particles scattered around the graphene which can be beneficial to build a high qualified conductive path.

## Data Availability

All data generated or analysed during this study are included in this published article (and its Supplementary Information files.
