# Author Correction: Effects of ultrasonic-assisted nickel pretreatment method on electroless copper plating over graphene

**DOI:** 10.1038/s41598-023-31841-z

**Published:** 2023-03-21

**Authors:** Qingyan Peng, Xiaodong Tan, Mohanapriya Venkataraman, Jiri Militky, Wei Xiong, Arunjunai Raj Mahendran, Herfried Lammer, Pavel Kejzlar

**Affiliations:** 1 Department of Material Engineering, Faculty of Textile Engineering, Technical University of Liberec, 461 17 Liberec, Czech Republic; 2 Hubei Key Laboratory of Biomass Fiber and Eco-Dyeing and Finishing, College of Chemistry and Chemical Engineering, Wuhan Textile University, Wuhan, 430073 China; 3 Wood K plus-Competence Center for Wood Composites and Wood Chemistry, Altenberger Straße 69, 4040 Linz, Austria; 4 Institute for Nanomaterials, Advanced Technologies and Innovation, Technical University of Liberec, 461 17 Liberec, Czech Republic

Correction to: *Scientific Reports* https://doi.org/10.1038/s41598-022-25457-y, published online 07 December 2022

In the original version of this Article, Wei Xiong was incorrectly listed as a corresponding author. The correct corresponding authors for this Article are Qingyan Peng and Mohanapriya Venkataraman. Correspondence and request for materials should be addressed to qingyan.peng@tul.cz or mohanapriya.venkataraman@tul.cz

Additionally, the order of the author names was incorrectly given as Qingyan Peng, Wei Xiong, Xiaodong Tan, Mohanapriya Venkataraman, Arunjunai Raj Mahendran, Herfried Lammer, Pavel Kejzlar & Jiri Militky.

Furthermore, the Author Contributions statement now reads:

“Conceptualization, Q.P., W.X., M.V.; data curation, Q.P., X.T., A.R.M., H.L.; formal analysis, Q.P., W.X.; resources. M.V., P.K.; software, Q.P., W.X., M.V. and X.T.; writing-original draft, Q.P. W.X. and X.T.; writing-review and editing, W.X., M.V., J.M. All authors reviewed the manuscript. All co-authors agree to publish the manuscript.”

Lastly, the Acknowledgements section contained an erroneous project grant number. The Acknowledgements now read:

“The work was supported by the project ‘Advanced structures for thermal insulation in extreme conditions’ (Reg. No. 21-32510 M) granted by the Czech Science Foundation (GACR). The authors also gratefully acknowledge Dr. Martin Stuchlík, Dr. Kaslik Josef for assistance in this work.”

The original Article has been corrected.

